# The impact of exercise self-efficacy on depression in firefighters: mediating effect of basic psychological needs and moderating effect of perceived social support

**DOI:** 10.3389/fpsyg.2025.1542883

**Published:** 2025-08-19

**Authors:** Wenjia Chen, Jiayi Yao, Guoqing Zhu, Zongyu Liu, Haozhe Wang, Dengshan Chu, Haitao Niu

**Affiliations:** ^1^School of Physical Education, China University of Mining and Technology, Xuzhou, China; ^2^School of Safety Engineering, China University of Mining and Technology, Xuzhou, China; ^3^Department of Sport and Exercise Science, College of Education, Zhejiang University, Hangzhou, China; ^4^School of Physical Education, Shandong University, Jinan, China

**Keywords:** firefighters, exercise self-efficacy, depression, basic psychological needs, perceived social support

## Abstract

**Background:**

Firefighters are exposed to high-stress work environments and are prone to depression, which has a significant impact on their professional performance and quality of life. Although exercise self-efficacy has been suggested to potentially influence psychological wellbeing, its mechanism of action on firefighters' depression has not been clarified.

**Methods:**

This study investigated the relationship between exercise self-efficacy and depression in firefighters and examined the mediating role of basic psychological needs and the moderating role of perceived social support. A cross-sectional research design was used to survey 450 Chinese frontline firefighters (mean age = 24.03 ± 5.27 years). Data were collected using the Exercise Self-Efficacy Scale (SES), the General Scale of Satisfaction with Basic Needs (BNSG-S), Patient Health Questionnaire-9 (PHQ-9), and Perceived Social Support Scale (PSSS). Descriptive statistics, correlation analyses, and moderated mediated effects analyses were performed using SPSS 22.0 and Hayes' PROCESS macro.

**Results:**

Exercise self-efficacy significantly and negatively predicted depression levels after controlling for demographic variables (β = −0.249, *p* < 0.01). Basic psychological needs partially mediated the association between exercise self-efficacy and depression (indirect effect β = −0.131, 95% CI: [−0.174, −0.096]), accounting for 52.61% of the total effect. Perceived social support moderated the direct association between exercise self-efficacy and depression, which was stronger at high levels of perceived social support (β = −0.234, 95% CI: [−0.365, −0.104], *p* < 0.001).

**Conclusions:**

This study reveals the mechanisms by which exercise self-efficacy influences firefighter depression levels through direct and indirect pathways, highlighting the critical role of basic psychological needs and perceived social support. These findings provide a theoretical basis for the development of targeted mental health intervention strategies for firefighters, emphasizing the importance of improving exercise self-efficacy, meeting basic psychological needs, and enhancing social support.

## 1 Introduction

Firefighters play a crucial role in preventing and mitigating major security risks and resolving major security risks and responding to and disposing of various types of disasters (Smith et al., 2016). As one of the most complex and dangerous occupations in society (Kales et al., 2007), the mental health of firefighters as a group warrants significant attention. The unique nature of their profession exposes firefighters to a high-pressure, high-intensity, and high-stress working environment for extended periods (Katsavouni et al., 2016), making them susceptible to various psychological problems due to their high perceived stress (Harvey et al., 2016). Stanley et al. (2016) found that the prevalence of suicidal thoughts and behaviors among firefighters is significantly higher than that of the general population. The mental health of firefighters in China is also concerning and necessitates urgent attention (Chen et al., 2020). Therefore, it is of utmost importance to focus on firefighters' depression and investigate the influencing mechanisms and intervention strategies to promote their physical and mental wellbeing.

Depression is a prevalent negative emotional state that can intensify an individual's experience of sadness (Rottenberg, 2005), impair moral cognitive ability (Szekeres and Wertheim, 2015), and is even closely related to extreme behaviors such as suicide (Conner et al., 2009). In their daily work, firefighters frequently encounter a wide range of emergencies and high-intensity rescue tasks. The immense pressure they face leads to negative emotions that are difficult to resolve and release in a timely manner, rendering them more susceptible to negative emotions, including depression (Sawhney et al., 2018). Depression diminishes firefighters' quality of life, causing insomnia, reduced work efficiency, and other issues, thereby significantly impacting their level of mental health (Carey et al., 2011).

Research has shown that not all firefighting tasks have the same impact on the mental health of firefighters. Harvey et al. (2016) found in a study of 753 firefighters from the Fire and Rescue Service in Australia that repeated exposure to traumatic events involving serious injuries and fatalities was the main predictor of depressive symptoms, with an incidence rate of 5%. Retired firefighters reported significantly higher levels of symptomatically, with depression accounting for 18% (*p* < 0.001). There is a significant positive correlation between the number of fatal accidents and depression. The systematic review by Stanley et al. (2016) found that firefighters who have experienced colleague injury incidents have a significantly increased risk of developing depressive symptoms. A survey of 220 firefighters found that 14% reported moderate to severe depression symptoms (Jones et al., 2018). Cross regional studies have shown that specific tasks exhibit specific responses to the impact on the mental health of firefighters. For example, in drug overdose rescue missions, 97% of firefighters experience at least one potential traumatic event (PTE), and the frequency of PTE exposure is significantly positively correlated with the degree of psychological distress (Wiegand et al., 2024). Tasks related to death events increase the risk of PTSD for firefighters by 3.2 times (Verble et al., 2024). These data indicate that specific types of high-intensity, traumatic tasks have a significantly greater negative impact on the mental health of firefighters.

Exercise self-efficacy refers to an individual's confidence and certainty in their ability to successfully execute and persist in exercise behaviors (Bandura, 1977). It not only includes confidence in starting to exercise, but also involves assessing the ability to persist in exercising when facing obstacles. This concept is different from general self-efficacy, as it is specific to the field of exercise and is a key psychological mechanism for predicting whether individuals will adopt and maintain exercise behavior (Lee et al., 2009; Murray et al., 2012). Numerous studies have confirmed that exercise can be effective in improving mental health and alleviating depressive symptoms (Xie et al., 2021; Pickett et al., 2017). Craft et al. (2008). demonstrated that exercise self-efficacy and social support might indirectly affect depressive symptoms through increased physical activity, supporting the mediator model of exercise self-efficacy in improving depression. For the firefighter community, Leary et al. (2020) found that a comprehensive exercise plan combining strength training and aerobic exercise can best improve their exercise self-efficacy and mental health level. Especially, functional training that simulates firefighting tasks such as weight-bearing climbing, dragging, and handling can not only enhance job-related skills, but also improve confidence and the ability to cope with stress (Zuckerman et al., 2021; Holland-Winkler et al., 2023). Research has shown that not all sports activities have the same effect on improving the mental health of firefighters. Craft et al. (2008) found that moderate-intensity exercise (50% peak oxygen uptake) significantly improved depressive mood compared to low-intensity (20% peak oxygen uptake) and high-intensity (80% peak oxygen uptake) exercise (*p* < 0.05). Moderate intensity exercise can immediately improve depressive mood (*d* = 0.72) and the effect lasts for at least 30 min (*d* = 0.65). For firefighters, a combination of 3–5 times a week, 30–45 min of aerobic exercise (such as running) and resistance training (such as weightlifting) is most effective (Jahnke et al., 2016). High intensity interval training (HIIT) is particularly suitable for firefighters' intense work pace due to its high efficiency (Chizewski et al., 2021). The special training system for Chinese firefighters provides practical evidence. Through high-intensity compound training such as weight-bearing running, climbing, and rapid descent, improve cardiovascular function and psychological resilience; Based on facilities such as smoke and heat rooms and ruins, simulate scenarios such as fire scene destruction and hazardous chemical disposal. Through team collaboration tasks, strengthen skills and pre adapt to the emotional impact of real rescue in high response scenarios. At the same time, the best exercise program designed for firefighters should be diverse, including aerobic, strength, flexibility, and team activity elements, while considering their work characteristics and personal preferences to maximize exercise persistence and mental health benefits. In summary, this study proposes Hypothesis 1: Exercise self-efficacy negatively predicts firefighters' depression levels.

Self-determination theory posits that autonomy, competence, and belonging are three basic psychological needs of humans (Ryan and Deci, 2000). Exercise self-efficacy is closely related to the fulfillment of these basic psychological needs. Firstly, the level of self-efficacy reflects the degree to which an occupation satisfies an individual's need for autonomy (Federici, 2013). Secondly, self-efficacy itself represents the fulfillment of the need for competence (Andiani and Jayanagara, 2023); Lastly, self-efficacy is positively related to the need for belonging (Hajek and König, 2017). Satisfaction of basic psychological needs can enhance an individual's vigor and wellbeing, whereas thwarted needs can lead to negative outcomes such as depression (Martela et al., 2023). One study found that the specific psychological need of firefighters to feel safe was negatively correlated with depressive symptoms (Wang et al., 2023). Sylvester et al. (2018) emphasized that the using basic psychological needs as mediators or moderators between psychological variables better reveals their significance for individual development. Based on these analyses, this study proposes Hypothesis 2: Basic psychological needs mediate the relationship between exercise self-efficacy and firefighter depression.

Social support refers to the various forms of help and support that individuals receive from their social network, including instrumental, informational and emotional support (Barrera Jr, 1986). Perceived social support emphasizes an individual's subjective feeling and evaluation of the support received, which has a positive impact on psychological health (Lakey and Orehek, 2011). Cohen and Wills proposed the buffering effect hypothesis of social support, which posits that perceived social support can alleviate the negative impact of stressful events on physical and mental health, thus playing a protective role in maintaining psychological wellbeing (Cohen and Wills, 1985). Firefighters in high-pressure work environments have a greater need for support from organizations and others to alleviate psychological stress (Eryilmaz et al., 2024). Perceived social support can promote individuals' participation in physical activity by increasing levels of exercise self-efficacy and motivation (Moya et al., 2021). Synthesizing established research, this study proposes Hypothesis 3: Perceived social support positively moderates the direct relationship between exercise self-efficacy and firefighter depression.

**Figure 1:**
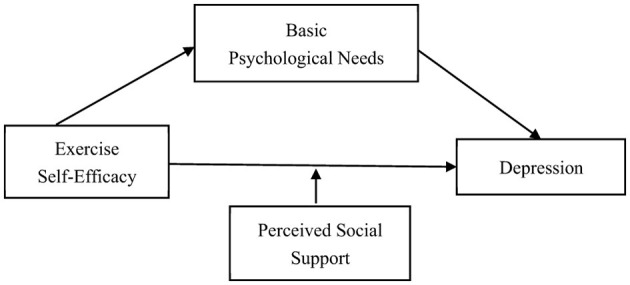
Research hypothesis model.

In summary, from the perspective of positive psychology, this study takes the influencing factors of depression as the entry point, integrating exercise self-efficacy theory, self-determination theory, and social support theory to construct a theoretical model that includes mediating effects and moderating roles (as shown in Figure 1). By examining the mechanisms of exercise self-efficacy, basic psychological needs, and perceived social support on firefighters' depression, this study aims to provide new theoretical perspectives and practical insights to alleviate the mental health problems experienced by the firefighter occupational group.

## 2 Study objects and methods

### 2.1 Participants and procedures

This study employed a cross-sectional design, and seven provinces (Beijing, Shanghai, Inner Mongolia, Hebei, Henan, Zhejiang, and Guangdong) out of 31 provincial-level administrative regions in China were selected as survey sites in July 2024 through a stratified whole-cluster random sampling method. The inclusion criteria were (1) first-line in-service firefighters and (2) voluntary participation in the study. The exclusion criteria included (1) inability to provide informed consent, (2) incomplete questionnaire responses, and (3) suspected careless responding based on completion time screening. The study utilized Wenjuanxing (Sojump), a professional online survey platform that provides comprehensive questionnaire establishment, distribution, management, collection, and analysis services with built-in data quality control features. An electronic questionnaire containing measures of the four main variables was created on the platform and generated as a QR code link for distribution.

The survey was distributed from July 15 to July 31, 2024, through a coordinated approach involving fire department administrators in each province. Research coordinators in each target province were provided with the survey QR code and standardized instructions. The QR codes were distributed to firefighters through official departmental WeChat groups and bulletin boards at fire stations. Research coordinators tracked the distribution process and monitored response rates in real-time through the Wenjuanxing platform's dashboard. A total of 503 questionnaires were distributed across the seven provinces. All 503 distributed surveys were accessed and completed (initial completion rate: 100%). To ensure data quality, we implemented multiple screening criteria: (1) completion time screening—based on the questionnaire length (52 items) and normal reading speeds, we set the minimum reasonable completion time at 200 s (~3.3 min) to prevent speeding and careless responding (Meade and Craig, 2012); (2) completeness screening—responses with missing data on key variables were excluded. After applying these quality control measures, 53 responses were excluded (47 for completion time < 200 s, 6 for incomplete data), resulting in 450 valid responses and a final valid response rate of 89.46%. All participants were informed of the study's purpose, procedures, and their right to withdraw at any time through an online information sheet. Electronic informed consent was obtained through the platform's built-in consent function. Researchers received uniform data collection training to ensure standardization, and research coordinators were available to answer participants' questions throughout the data collection period. The study was approved by the Ethics Committee of Shandong University (Approval No. 2021-1-115).

### 2.2 Research tools

(1) The Exercise Self-efficacy Scale (SES).

This study adopted the General Self-Efficacy Scale (SES), developed by Zhang and Hasibagen (2022). The 10-item scale assesses the degree of confidence in adhering to exercise in different situations. It using an 11-point scoring system (0–10), ranging from “no confidence at all” to “very confident”. A higher total score indicates greater exercise self-efficacy. The Cronbach's α coefficient of this scale in this study was 0.969.

(2) The General Scale of Satisfaction with Basic Needs (BNSG-S).

The study adopts the Chinese version of the General Scale of Satisfaction with Basic Needs (BNSG-S) of Deci and Ryan (2000). This scale includes three dimensions: autonomy, competence and relatedness, with a total of 21 items. Each item uses a 7-point Likert scale (1 = completely inconsistent, 7 = completely consistent). The higher the score, the higher the degree of satisfaction of basic psychological needs. In this study, the Cronbach's α coefficient of this scale is 0.948.

(3) Patient Health Questionnaire-9 (PHQ-9).

This study used Patel et al. (2019) to design and validate PHQ-9 according to the criteria of the Diagnostic and Statistical Manual of Mental Disorders (DSM-IV). This scale consists of 9 items, each corresponding to a core symptom of depression in DSM-IV. The PHQ-9 score ranges from 0 to 27 points, with higher scores indicating more severe depressive symptoms. According to the scoring results, depressive symptoms can be divided into no depression (0–4 points), mild depression (5–9 points), moderate depression (10–14 points), and severe depression (≥15 points). The Cronbach's alpha coefficient of the scale in this study is 0.945.

(4) Perceived Social Support Scale (PSSS).

This study adopts the scale compiled by Blumenthal et al. (1987). The scale contains 12 questions. The questions directly related to social support are divided into three dimensions of family, friends, and important others according to the source of support. Each dimension consists of four questions. This scale uses a scoring system ranging from 1 (strongly disagree) to 7 (strongly agree). A higher total score indicates a higher individual social support. The Cronbach's α coefficient of this scale in this study was 0.978.

### 2.3 Statistical method

All data analyses in this study were completed using SPSS 26.0. Statistical significance was determined using conventional alpha levels: *p* ≥ 0.05 was considered non-significant, *p* < 0.05 was considered statistically significant, and *p* < 0.001 was considered highly statistically significant. All hypothesis testing employed an alpha level of 0.05. First, the Harman single-factor analysis method was used to test for common method variance. Principal component analysis extracted five factors with eigenvalues >1. The explanatory power of the first factor was 28.79% (< 40%), indicating that there was no serious problem of common method variance. Prior to conducting correlation analyses, normality assumptions were assessed through multiple approaches including Shapiro-Wilk tests, examination of skewness and kurtosis values, and visual inspection of Q-Q plots. Formal normality tests indicated significant deviations from perfect normality for all variables (all *p* < 0.001). However, examination of skewness and kurtosis revealed that most variables demonstrated acceptable departures from normality, with values generally within or close to conventional thresholds (skewness < 2, kurtosis < 2). Specifically, skewness ranged from −0.730 to 1.369, and kurtosis ranged from −0.485 to 1.348. Given the large sample size (*N* = 450), Pearson product-moment correlations were employed based on the robustness of correlation analysis to moderate normality violations in large samples. To validate this approach, Spearman rank-order correlations were computed as a robustness check and yielded highly consistent patterns with Pearson correlations (maximum difference = 0.103), confirming the appropriateness of the parametric approach. Second, descriptive statistics and correlation analyses were conducted on each variable. Furthermore, Model 4 and Model 59 (Hayes, 2017) of the PROCESS macro were used to test the mediation effect of hopelessness and the moderation effect of psychological stress, respectively. The significance of mediating and moderating effects was determined by the 95% confidence interval (CI) using the Bootstrap method (repeated sampling 5,000 times). When the CI did not contain 0, the effect was considered significant. Before constructing the mediation model, all variables were standardized, and demographic factors such as work location and highest education were included as covariates to control for potential confounding effects.

## 3 Results

### 3.1 Descriptive statistics and correlation analysis

A total of 450 firefighters' data were analyzed in this study. The average height of the subjects was 173.77 ± 80.74 cm and the average weight was 88.09 ± 29.10 kg. The mean age of the subjects was 24.03 ± 5.27 years and the mean years of service was 6.300 ± 4.903 years. In terms of marital status, 140 (31.11%) were unmarried, 282 (62.67%) were married, 23 (5.11%) were divorced, and 6 (1.11%) were remarried. The distribution of highest educational attainment was as follows: 168 (37.33%) were below junior college, 202 (44.89%) were college, 79 (17.56%) were bachelor's degree, and 1 (0.22%) was master's degree. Regarding the work location, 155 (34.44%) worked in rural areas and 295 (65.56%) in urban areas (Table 1).

**Table 1:** Demographic characteristics of study participants.

**Characteristics**	**Category**	**Total (*n =* 450)**
Height (cm)		173.77 ± 80.74
Weight (kg)		88.09 ± 29.10
Age (years)		24.03 ± 5.27
Years of service (years)		6.30 ± 4.90
Marital status, *n* (%)	Unmarried	140 (31.11%)
	Married	282 (62.67%)
	Divorcee	23 (5.11%)
	Remarry	6 (1.11%)
Highest education *n* (%)	Less than tertiary	168 (37.33%)
	Three-year college	202 (44.89%)
	Undergraduate (adjective)	79 (17.56%)
	Master's degree student	1 (0.22%)
Work location *n* (%)	countryside	155 (34.44%)
	municipalities	295 (65.56%)

Data were described as n (%) or mean ± SD.

**Figure 2:**
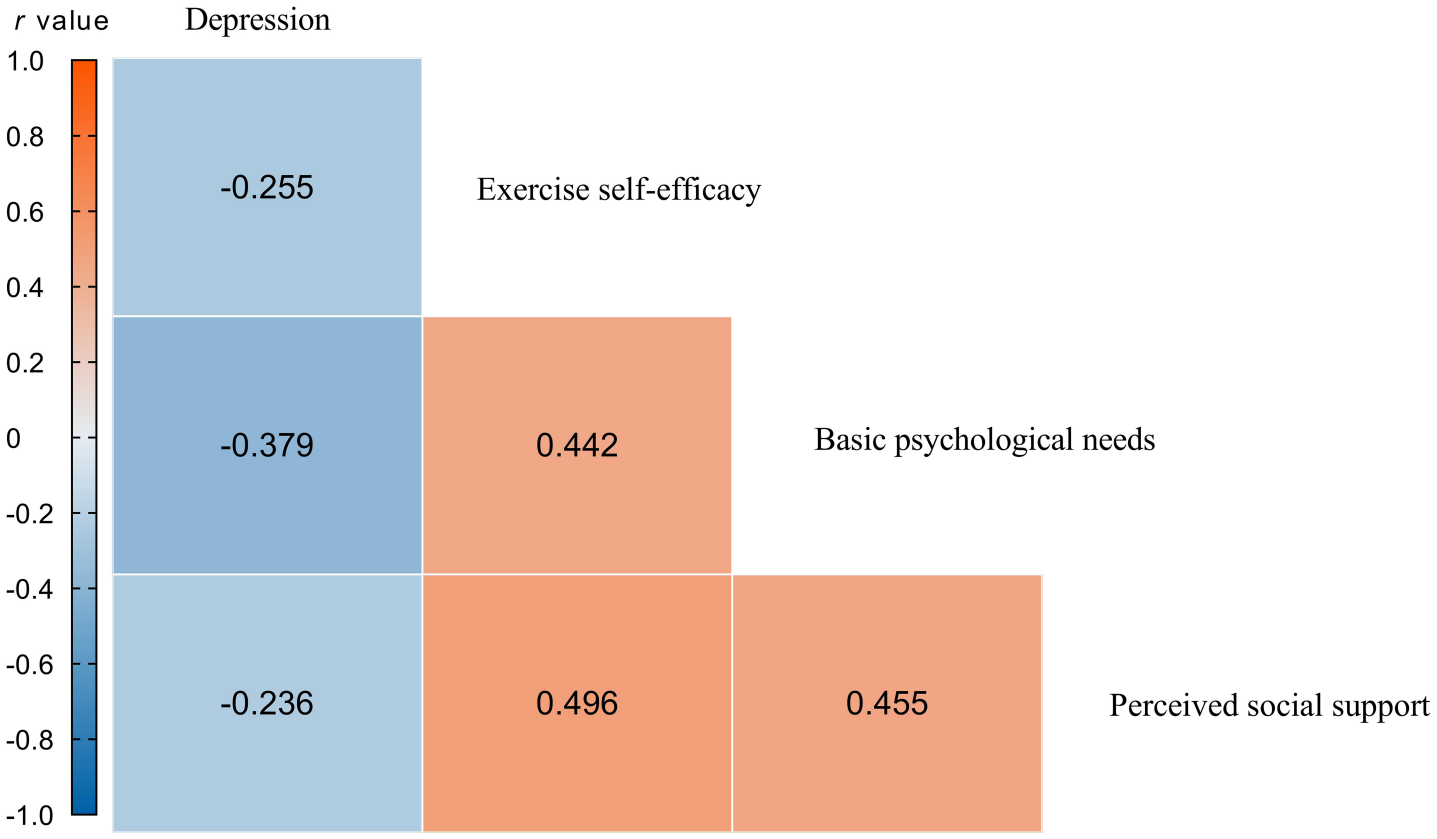
Pearson correlation coefficient result. **Blue**, negative correlations; **red**, positive correlations.

As shown in Figure 2, firefighters' exercise self-efficacy was significantly and positively correlated with basic psychological needs (*r* = 0.442, *p* < 0.01), perceived social support (*r* = 0.496, *p* < 0.01), and significantly and negatively correlated with depression (*r* = −0.255, *p* < 0.01); basic psychological needs, perceived social support, and depression were all significantly and negatively correlated (*r* =−0.379, *p* < 0.01; *r* = −0.236, *p* < 0.05). The correlations between the variables were in line with theoretical expectations, providing a preliminary basis for the analysis of mediating and moderating effects.

### 3.2 Tests of the mediating effect of basic psychological needs

Model 4 of the PROCESS macro was used to test the mediating effect of basic psychological needs between exercise self-efficacy and depression. After controlling for demographic variables, the results (Table 2) showed that exercise self-efficacy significantly positively predicted basic psychological needs (β = 0.428, *p* < 0.01), where β represents the standardized regression coefficient and p indicates the significance level, while significantly negatively predicting depression (β = −0.249, *p* < 0.01). After controlling for exercise self-efficacy, basic psychological needs significantly negatively predicted depression (β = −0.305, *p* < 0.01) and the absolute value of the coefficient of prediction of depression by exercise self-efficacy decreased.

**Table 2:** Test table for the mediating effect model of exercise self-efficacy.

**Independent variables**	**Model 1 (Depression)**		**Model 2 (Basic Psychological Needs)**		**Model 3 (Depression)**	
	β	**t**	β	**t**	β	**t**
Age	0.066	0.906	−0.100	−1.489	0.036	0.507
Marital status	0.021	0.353	0.009	0.166	0.024	0.416
Highest education	0.030	0.653	0.091	2.140^*^	0.058	1.298
Years of service	0.130	1.755	−0.116	−1.695	0.095	1.327
Work location	−0.048	−1.063	0.119	2.847^**^	−0.012	−0.273
Exercise self-efficacy	−0.249	−5.535^**^	0.428	10.334^**^	−0.119	−2.463^*^
Basic psychological needs					−0.305	−6.144^**^
*R* ^2^	0.108		0.247		0.178	
*F*	8.948^**^		24.278^**^		13.699^**^	

Significance levels: ^*^ p < 0.05, ^**^ p < 0.01. β represents standardized regression coefficients. t represents t-test statistics for significance testing of regression coefficients.

Further combining the Bootstrap results (Table 3) shows that exercise self-efficacy had a significant indirect effect on depression through basic psychological needs (β = −0.131, 95% CI = [−0.174, −0.096]), where CI represents the confidence interval, which accounted for 52.61% of the total effect, and a significant direct effect (β = −0.119, 95% CI = [−0.213, −0.024]), accounting for 47.79% of the total effect. In summary, basic psychological needs partially mediated the effect of exercise self-efficacy on depression, and Hypothesis 2 was supported.

**Table 3:** Mediating effects of basic psychological needs between exercise self-efficacy and depression.

**Effect types**	**Path**	**95% CI**	**Effect**
Direct effect	Exercise Self-Efficacy > Depression	[−0.213, −0.024]	−0.119
Indirect effect	Exercise Self-Efficacy > Basic Psychological Needs > Depression	[−0.174, −0.096]	−0.131
Total effect	-	[−0.338, −0.161]	−0.249

Bootstrap 95% confidence intervals based on 5,000 resamples. Effects are significant when confidence intervals exclude zero.

### 3.3 Tests of the moderating effect of perceived social support

**Figure 3:**
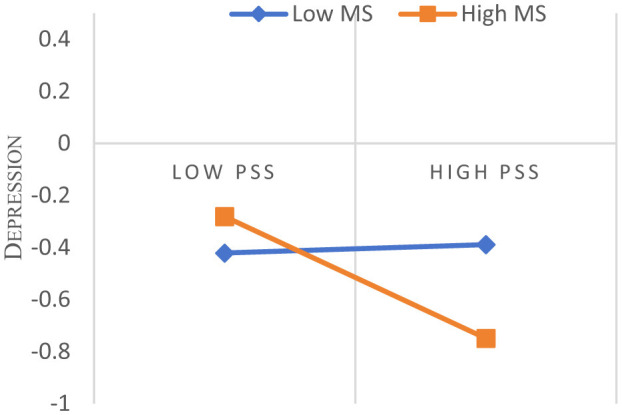
Perceived social support moderates exercise self-efficacy on depression. PSS, Perceived Social Support. Simple slopes plotted at ±1 SD of the moderator. Lower depression values indicate better mental health.

The mediation model with moderation was further tested using Model 59 of the PROCESS macro. After controlling for demographic variables, the results (Table 4) indicated that the interaction term between exercise self-efficacy and perceived social support significantly and negatively predicted depression (β = −0.125, *p* < 0.01), whereas the interaction term between basic psychological needs and perceived social support did not significantly predict depression (β = 0.046, *p* > 0.05). This suggests that perceived social support significantly moderated the direct relationship between exercise self-efficacy and depression, but not the relationship between basic psychological needs and depression (Figure 3).

**Table 4:** Mediation model test table with moderation.

**Independent variables**	**Depression**		**Basic psychological needs**	
	β	**t**	β	**t**
Age	0.035	0.634	−0.078	−1.528
Marital status	0.037	0.378	0.015	0.171
Highest education	0.079	1.313	0.093	1.672
Years of service	0.098	1.244	−0.099	−1.368
Work location	−0.044	−0.479	0.214	2.526^*^
Exercise self-efficacy	−0.109	−2.048^*^	0.286	6.221^**^
Perceived social support	−0.055	−1.044	0.281	6.045^**^
Exercise self-efficacy^*^ perceived social support	−0.125	−2.998^**^	−0.018	−0.551
Basic psychological needs	−0.319	−5.557^**^		
Basic psychological needs^*^ perceived social support	0.046	0.854		
*R* ^2^	0.197		0.307	
*F*	10.794		24.467	

Significance levels: ^*^p < 0.05, ^**^p < 0.01. β represents standardized regression coefficients. t represents t-test statistics for significance testing of regression coefficients.

To facilitate the interpretation of the moderating effect of perceived social support, the conditional direct effect of exercise self-efficacy on depression was examined at three levels of perceived social support: low (−1SD), medium (M), and high (+1SD), respectively. It was found that the direct effect of exercise self-efficacy on depression increased progressively from low to high levels of perceived social support: from non-significant (β = 0.016, SE = 0.069, 95% CI = [−0.119, 0.151], *p* = 0.815), to significant (β = −0.109, SE = 0.053, 95% CI = [−0.214, −0.005], *p* < 0.05), to highly significant (β = −0.234, SE = 0.067, 95% CI = [−0.365, −0.104], *p* < 0.001).

Conditional indirect effects of exercise self-efficacy on depression via basic psychological needs were also examined. The indirect effect was significant at all three levels of perceived social support, low, medium and high, but the strength of the effect diminished with increasing levels of perceived social support: β = −0.111 (SE = 0.037, 95% CI = [−0.193, −0.047]), β = −0.091 (SE = 0.023, 95% CI = [−0.141, −0.052], respectively), β = −0.073 (SE = 0.021, 95% CI = [−0.124, −0.038]).

## 4 Discussion

### 4.1 The relationship between exercise self-efficacy and depression in firefighters

The findings of this study indicate that exercise self-efficacy exhibits a significant negative correlation with firefighters' depression levels (*r* = −0.255, *p* < 0.01), suggesting that higher exercise self-efficacy is associated with lower levels of depression among firefighters. This discovery aligns with previous research (Lambert et al., 2012; Makara-Studzińska et al., 2019), corroborating that elevated exercise self-efficacy can directly mitigate firefighters' depression levels, thus supporting Hypothesis 1. This result can be elucidated from both neurobiology and mental health perspectives. From a neurobiological standpoint, exercise can enhance neurotransmitter synthesis and secretion, improve synaptic transmission efficiency, facilitate the release of neurotrophic factors, and augment gene expression, neurogenesis, and angiogenesis in brain regions associated with learning and memory, thereby enhancing cognitive abilities (Lin and Kuo, 2013). These biological mechanisms collectively contribute to the suppression of depression. From a mental health perspective, exercise can bolster self-confidence and self-esteem (Kim and Ahn, 2021), divert attention, provide social support, and consequently alleviate negative emotions (Thoits, 1995). Simultaneously, alterations in exercise behavior are accompanied by positive psychological changes (Fox, 1999; Bower et al., 2007), fostering the maintenance of a healthy mental and physical state.

In recent years, numerous studies have further substantiated the negative predictive role of exercise self-efficacy on depression. Goodwin et al. surveyed 5,877 participants and demonstrated that regular exercise is significantly associated with lower levels of depression (Goodwin, 2003). Subsequently, Harvey et al. (2010) conducted a study that controlled for other variables such as age and gender, revealing that participants' leisure-time exercise exhibited a significant negative correlation with depression and comorbid depression-anxiety. Miller et al. (2019) further corroborated that exercise behavior, exercise self-efficacy, and social support were all negatively correlated with depressive symptoms (*r* = −0.20 to −0.56). Similarly, Kangas et al. (2015) conducted a 4-week follow-up assessment of 116 physically inactive adults (35% reported clinically significant depressive symptoms), and the results indicated that exercise self-efficacy was significantly negatively correlated with depressive symptoms. The study emphasized the importance of targeting self-efficacy in exercise interventions, particularly for individuals with depressive symptoms. These studies have validated the positive impact of exercise self-efficacy on ameliorating depressive symptoms from various perspectives. Depression exerts a significant influence on the mental and physical health and job performance of individuals across various occupations (Adler et al., 2006). Firefighters, in particular, are at high risk for developing depression due to their frequent exposure to high-stress and hazardous work environments. Therefore, it is imperative to implement effective measures to enhance firefighters' exercise self-efficacy to mitigate their depression levels.

### 4.2 Mediating effects of basic psychological needs

The study results confirm hypotheses H2 and H3, demonstrating that basic psychological needs negatively predict firefighters' depression levels and play a mediating role in the relationship between exercise self-efficacy and firefighters' depression. Generally, the satisfaction of basic psychological needs is particularly crucial in the firefighter population. Firefighters are exposed to potentially traumatic events more frequently than the general population (Berger et al., 2012), often accompanied by heightened arousal or dissociation and feelings of helplessness (Geuzinge et al., 2020), which can undermine their basic psychological needs. Rouse et al. (2020) posit that when basic psychological needs are actively thwarted, firefighters' psychological dysfunction and ill-being become evident. Bartholomew et al. (2018) conducted a year-long longitudinal study and discovered that teachers' controlling teaching styles diminish students' sense of basic psychological need satisfaction, leading to more negative emotional experiences. This finding substantiates that the erosion of basic psychological needs may increase the likelihood of developing negative psychological disorders such as depression. Thus, hypothesis H2 is confirmed, indicating that the satisfaction of basic psychological needs is negatively correlated with firefighters' depression levels.

The study reveals that basic psychological needs play a mediating role in the impact of exercise self-efficacy on firefighters' depression, which is consistent with the findings of Richard et al. Specifically, higher levels of exercise self-efficacy facilitate the satisfaction of firefighters' basic psychological needs, thereby reducing the likelihood of depression (Ryan and Deci, 2020). Firefighters with high exercise self-efficacy often maintain a positive attitude and even exhibit psychological resilience when faced with difficulties and adversity (Ren et al., 2020). Further mediation effect tests demonstrate that after controlling for exercise self-efficacy, the predictive effect of basic psychological needs on depression remains significant (*B* = −0.305, *p* < 0.01). This indicates that basic psychological needs play a significant role in the process of exercise self-efficacy influencing depression. Exercise self-efficacy may indirectly reduce depression levels by satisfying basic psychological needs, thus confirming hypothesis H3. Therefore, governments and fire departments should implement measures to meet firefighters' basic psychological needs, such as providing psychological counseling and organizing sports activities, to promote their mental health development and improve work efficiency (Liu et al., 2022).

### 4.3 Moderating effects of perceived social support

This study reveals that perceived social support significantly moderates the direct relationship between exercise self-efficacy and firefighter depression. Specifically, when the level of perceived social support is higher, the negative impact of exercise self-efficacy on depression is stronger, supporting hypothesis H4. This result can be explained by the Stress Buffering Theory (SBT) (Wang et al., 2021). According to SBT, social support can reduce an individual's appraisal response to stressors, attenuate physiological stress responses, and thereby mitigate the negative impact of stress on health.

Firefighters are constantly exposed to high-pressure work environments, facing life-threatening situations and traumatic events, which can easily lead to mental health problems such as secondary traumatic stress disorder (STSD) and depression (Oda et al., 1988). In turn, these problems can undermine their ability to cope with stress (Geuzinge et al., 2020). The findings of this study indicate that perceived social support can alleviate depressive symptoms in firefighters by enhancing their exercise self-efficacy. The stress-buffering process may involve three aspects: (1) In terms of appraisal and coping, when individuals face stressful events, they engage in primary and secondary appraisals. Social support influences these two stages, reducing the perceived threat of the event and increasing the individual's sense of coping ability (Luria and Torjman, 2009). Social support also provides individuals with supportive resources, including problem-solving strategies, emotional comfort, and information provision. These resources help individuals adopt more effective coping strategies when facing stress, thereby mitigating the negative impact of stress on firefighters' physical and mental health (Cogan and MacDonald, 2021). (2) Regarding the internal cognitive system, social support influences an individual's perception and appraisal of stressful events. Individuals who receive social support tend to underestimate the harmfulness of stressful situations, reducing their assessment of the severity of stressful events by increasing their perceived self-coping ability, thus protecting the physical and mental health of individual firefighters (Pocnet et al., 2016). (3) In terms of dynamic effects, the dynamic effects model posits that social support and stress should be viewed as independent variables acting simultaneously on the level of physical and mental health (Pinxten and Lievens, 2014). They interact and influence each other, implying that the stress-buffering process through social support requires different strategies based on the firefighters' stress levels. Consequently, through the effect of social support, the physical and mental benefits of exercise are enhanced, and the adverse effects on individual physical and mental health are reduced. Simultaneously, it confirms the validity of hypothesis H1, which states that exercise self-efficacy can negatively predict firefighter depression.

Social support plays a positive role in alleviating depression, and integration into a supportive social network is crucial for the mental health of high-risk populations (Jia et al., 2015). Carpenter et al. (2015) study of 334 professional firefighters showed that good social support can mitigate the negative psychological impact of work stress. Bekiros et al. (2022) found that community-provided social support can reduce psychological distress and buffer the impact of COVID-19-related stressors. Furthermore, studies targeting high-pressure groups such as doctors (Linos et al., 2022), professional athletes (Delfin et al., 2024), and others have also confirmed the positive role of peer and family support in coping with various physical and mental stressors. Considering that firefighters, doctors, and athletes belong to high-intensity and high-pressure occupations, the above research findings may also apply to the firefighter population. Therefore, as an important moderating factor in the prevention and intervention of firefighter depression, perceived social support is of great significance for ensuring their physical and mental health and enhancing work efficiency.

### 4.4 Research limitations and prospects

This study explores the relationship between exercise self-efficacy and depression in firefighters, as well as the underlying mechanisms of basic psychological needs and perceived social support, providing a new theoretical perspective for understanding and intervening in firefighter depression from a positive psychology perspective. However, this study still has some limitations. Firstly, the cross-sectional study design and self-report questionnaire have limitations in causal inference and measurement bias. We used Harman's one-way analysis to evaluate the common method bias (CMB), but recent studies have shown that this method may not be sufficient to reliably distinguish between different study designs (Howard et al., 2024). Although this method is widely used in the field, its limitations may affect the accurate evaluation of CMB. Secondly, this study focuses on exercise self-efficacy rather than actual exercise behavior (type, duration, frequency), and the lack of measurement of specific exercise behavior limits our ability to understand the impact of different exercise types on mental health. In addition, although this study found that perceived social support regulates the indirect pathway through which exercise self-efficacy affects depression through basic psychological needs, its specific mechanism and boundary conditions need further exploration. Finally, this research model needs to incorporate more contextual and individual factors to construct a more comprehensive model of the impact mechanism of firefighters' mental health, and to test its cross industry applicability.

Future research should adopt longitudinal or experimental designs, combined with objective measurement indicators to verify the stability and causal relationship of the results; Consider combining more advanced statistical methods, such as marker variable method or direct measurement method, with various strategies to comprehensively control and evaluate CMB; Simultaneously measuring exercise self-efficacy and actual exercise behavior, exploring the differentiated effects of different types of exercise on mental health. It is particularly noteworthy that we recommend future research to focus on the female firefighter population through purposeful sampling strategies or multi country cooperation, in order to obtain sufficient sample size for gender comparative analysis. This will provide valuable insights into potential gender differences in the relationships we are studying. In summary, this study has preliminary revealed the psychological mechanism by which exercise self-efficacy affects depression in firefighters. However, further improvements are needed in research design, mechanism exploration, and theoretical expansion to provide scientific guidance for more effective promotion of firefighters' physical and mental health.

## 5 Conclusion

In conclusion, this study delved into the intricate relationships between exercise self-efficacy, basic psychological needs satisfaction, perceived social support, and depression among Chinese firefighters, while simultaneously unraveling the underlying mechanisms of basic psychological needs and perceived social support within these complex associations. The findings illuminate three key points: (1) exercise self-efficacy exhibits a significant negative correlation with firefighters' depression levels, underscoring the potential of enhancing exercise self-efficacy as an effective means to mitigate the risk of depression; (2) basic psychological needs serve as partial mediators in the relationship between exercise self-efficacy and firefighters' depression, suggesting that exercise self-efficacy can indirectly alleviate depressive symptoms by fulfilling firefighters' fundamental psychological needs; (3) perceived social support significantly moderates the direct relationship between exercise self-efficacy and firefighters' depression, with higher levels of perceived social support amplifying the beneficial impact of exercise self-efficacy on reducing depressive symptomatically.

## Data Availability

The raw data supporting the conclusions of this article will be made available by the authors, without undue reservation.
